# Alternariol Monomethyl-Ether Induces Toxicity via Cell Death and Oxidative Stress in Swine Intestinal Epithelial Cells

**DOI:** 10.3390/toxins16050223

**Published:** 2024-05-11

**Authors:** Daniela Eliza Marin, Valeria Cristina Bulgaru, AnaMaria Pertea, Iulian Alexandru Grosu, Gina Cecilia Pistol, Ionelia Taranu

**Affiliations:** National Research and Development Institute for Biology and Animal Nutrition (INCDBNA-IBNA-Balotesti), Calea Bucuresti nr.1, 077015 Balotesti Ilfov, Romania; cristina.bulgaru@ibna.ro (V.C.B.); ana.pertea@ibna.ro (A.P.); grosu.iulian@ibna.ro (I.A.G.); gina.pistol@ibna.ro (G.C.P.); ionelia.taranu@ibna.ro (I.T.)

**Keywords:** alternariol monomethyl-ether, intestinal epithelium, swine, apoptosis, oxidative stress

## Abstract

Alternariol monomethyl-ether (AME), together with altenuene and alternariol, belongs to the *Alternaria* mycotoxins group, which can contaminate different substrates, including cereals. The aim of the present study was to obtain a deeper understanding concerning the effects of AME on pig intestinal health using epithelial intestinal cell lines as the data concerning the possible effects of *Alternaria* toxins on swine are scarce and insufficient for assessing the risk represented by *Alternaria* toxins for animal health. Our results have shown a dose-related effect on IPEC-1 cell viability, with an IC50 value of 10.5 μM. Exposure to the toxin induced an increase in total apoptotic cells, suggesting that AME induces programmed cell death through apoptosis based on caspase-3/7 activation in IPEC-1 cells. DNA and protein oxidative damage triggered by AME were associated with an alteration of the antioxidant response, as shown by a decrease in the enzymatic activity of catalase and superoxide dismutase. These effects on the oxidative response can be related to an inhibition of the Akt/Nrf2/HO-1 signaling pathway; however, further studies are needed in order to validate these in vitro data using in vivo trials in swine.

## 1. Introduction

*Alternaria* toxins are secondary metabolites with different chemical structures, which are produced by fungus belonging to *Alternaria* species [1,2]. More than 250 secondary metabolites are produced by *Alternaria* toxins, but only some of them are characterized and reported as mycotoxins [3]. *Alternariol* monomethyl-ether (AME), together with altenuene and alternariol, belongs to the *Alternaria* mycotoxins group of dibenzo-pyrone derivatives, which can contaminate different substrates, including cereals. The contamination of cereals with mycotoxins represents a worldwide concern, as cereals represent an important constituent of human and animal diets [4]. AME was detected in 23% of wheat samples analyzed in Argentina between 2004 and 2005, with a maximum concentration of 7451 μg/kg and a mean concentration of 2118 μg/kg [5]. Other studies analyzing the occurrence of *Alternaria* toxins in wheat samples from China have indicated the presence of AME in 21 samples from a total of 22, with a maximum concentration of 1426 μg/kg and a mean concentration of 443 μg/kg [6]. According to EFSA, the mean concentration of AME in cereals ranges between 1.1 and 11 μg/kg [7]; however, higher concentrations (up to 184 μg/kg) were found in samples of cereals analyzed in Sweden [1]. Moreover, a multi-mycotoxin screening of European feed samples revealed that 82% of the analyzed samples were contaminated with AME, with a maximum concentration of 733 μg/kg and a mean concentration of 1.4 μg/kg [8]. Pigs, through the consumption of a diet rich in cereals, are particularly exposed to mycotoxin contamination [9]. Indeed, a survey on fungal secondary metabolites in pig feed samples carried out between 2014 and 2019 indicated that 40.3% of samples were contaminated with AME, with a maximum concentration of 208 μg/kg [10]. Exposure to *Alternaria* mycotoxins was associated with different negative effects on human and animal health, including cytotoxic, mutagenic, genotoxic, and carcinogenic effects [7,11,12]. Even if acute AME toxicity is low, AME exposure can induce gene mutations, chromosome breakage, and DNA damage, as demonstrated by in vivo and in vitro experiments [13,14]. Moreover, AME exposure is linked to certain forms of cancer, such as esophageal cancer. After oral ingestion, mycotoxins present in food or feed reach the body and interact with the cells of the gastrointestinal tract [15]. The intestinal epithelium realizes a physical barrier that separates the environment and the body in order to limit the passage of harmful antigens and microorganisms to other tissues and organs whilst ensuring that nutrients and water are absorbed [16]. Mycotoxins are absorbed and metabolized at an intestinal level before reaching systemic circulation or being excreted through feces [17]. Using a Caco-2 system in vitro and based on apparent permeability coefficients values, Burkhardt and collaborators demonstrated poor absorption of AME by intestinal epithelial cells compared to AOH [18]. A study that investigated the metabolism and excretion of *Alternaria* toxins in rats showed that they displayed a high fecal excretion rate (88%) of AME, which was much higher than the urinary excretion rate that was found (6–10%) [19]. AME exhibits cytotoxic effects on Caco-2 cells, with EC50 values between 6 and 23 μg/mL [20]. To the best of our knowledge, there are no studies concerning the effects of *Alternaria* toxins in swine, especially concerning their toxic effects on gut health.

The use of cell lines for toxicology studies is very important in the context of reducing the use of animals in trials, as suggested in the 3Rs principles [21]. Although the results from in vitro experiments using cell lines only partially reflect what happens in vivo, the cell models can provide important information concerning the cytotoxicity and mode of actions of toxicants [22]. For example, the Caco2 human intestinal epithelial cell line is widely used as a model of the intestinal epithelial barrier for assessing the effects of toxicants at a gut level [17,23]. Similarly to Caco2 cells, porcine intestinal epithelial cell line IPEC-1 cells also have the capacity to differentiate into a polarized cell monolayer with tight junctions, which can partially simulate the physiological processes of the small intestine’s epithelium [24]. The aim of the present study was to obtain a deeper understanding concerning the effects of AME on pig intestinal health using a porcine IPEC-1 cell line as the data concerning the possible effects of *Alternaria* toxins on swine are scarce and insufficient for assessing the risk represented by *Alternaria* toxins for animal health. As intestinal epithelial cells are continuously renewed, we used proliferating IPEC-1 cells as a realistic cell model for assessing AME exposure in our study.

## 2. Results

### 2.1. Effect of AME Exposure on IPEC-1 Cell Proliferation, Apoptosis, and Necrosis

**Figure 1:**
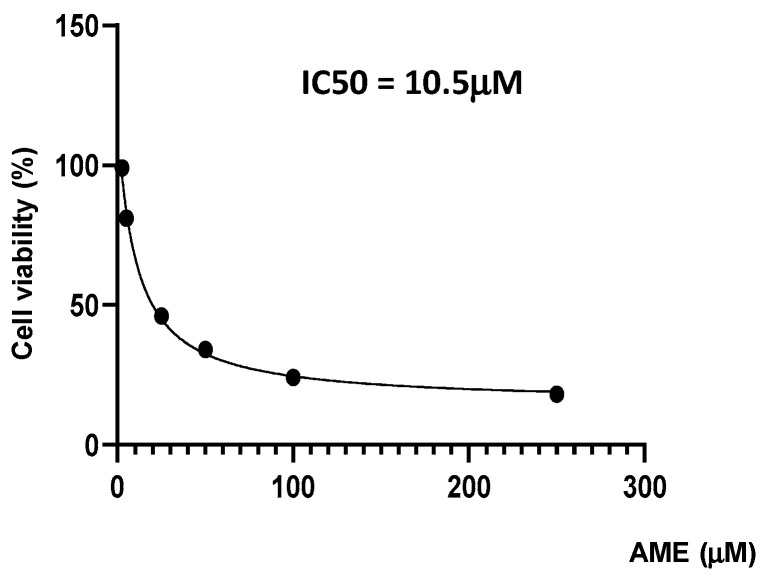
Effect of AME mycotoxin on cell viability. IPEC-1 cells were treated or left untreated for 24 h with different concentrations of AME, and the cytotoxic effect of the toxin was analyzed using an MTT assay. The IC50 value was calculated using GraphPad Prism 9 software. Data are presented as percentages of cell viability and represent the mean ± SEM of three independent experiments.

Firstly, we investigate the effects of the toxin on cell proliferation using an MTT assay. Figure 1 indicates a dose-related effect of the toxin on cell proliferation with a calculated IC50 of 10.5 μM.

**Figure 2:**
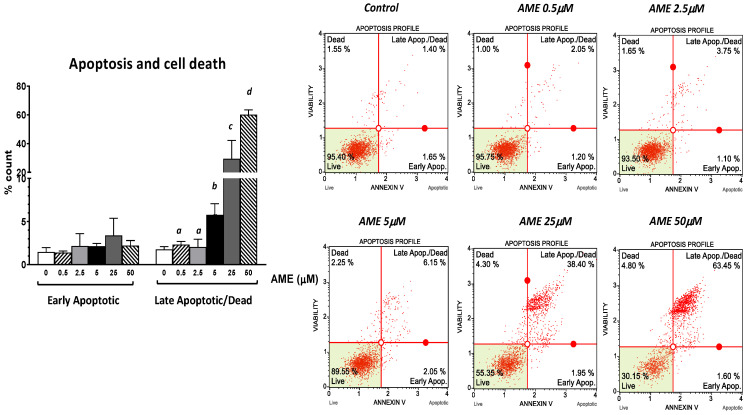
Effect of AME mycotoxin on cell viability, apoptosis, and cell death. IPEC-1 cells were treated or left untreated for 24 h with different concentrations of AME, and an apoptosis profile was performed using a Muse Annexin and Dead Cell kit, according to the manufacturer’s instructions. Data are presented as a percentage of live, early/late apoptotic, or dead cells and represent the mean ± SEM of three independent experiments; ^a,b,c,d^ show significant differences between different groups (*p* < 0.05).

Next, we assessed the effect of AME on cell proliferation, apoptosis, and necrosis using flow cytometry. As shown in Figure 2, the exposure of intestinal epithelial cells IPEC-1 to low and medium concentrations of AME (0.5, 2.5, and 5 μM) for 24 h have little or no effect on cell viability. Regardless of how much the concentration of AME increases, the cytotoxic effect stays dose-dependent. Thus, a concentration of 25 μM significantly decreased cell viability by 38.85%, while a concentration of 50 μM induced a decrease in cell viability by 67.03% compared to the untreated control. Exposure of IPEC-1 cells to AME has no effects on the early stages of apoptosis, but it increases the percentage of cells in late apoptosis or dead cells at concentrations higher than 5 μM: by 5.76% for a concentration of 5 μM (*p* = 0.060); by 29.4% for a concentration of 25 μM (*p* = 0.025); and by 60.16% for a concentration of 50 μM (*p* < 0.0001) compared to the control (1.76%).

### 2.2. Effect of AME Exposure on IPEC-1 Cell Cycle

**Figure 3:**
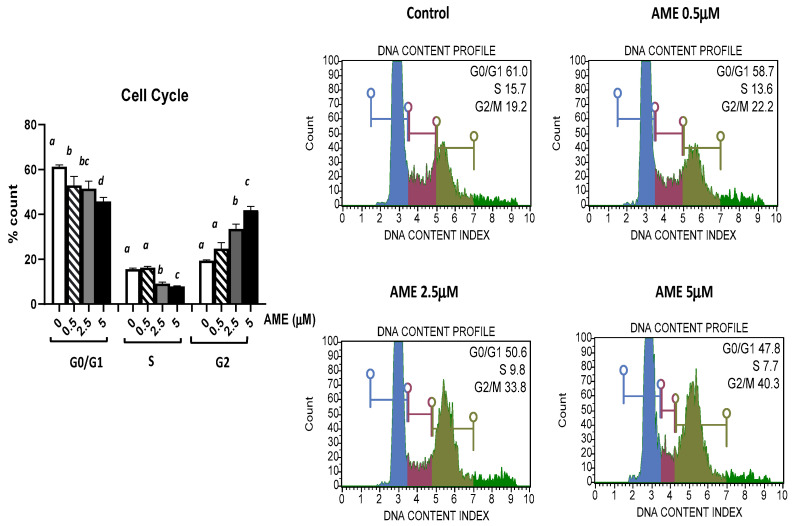
Effect of AME mycotoxin on cell cycle. IPEC-1 cells were treated or left untreated for 24 h with different concentrations of AME, and cell cycle analysis was performed using a Muse Cell Cycle Analysis kit, following the manufacturer’s instructions. Data for cell cycle analysis were expressed as the percentage of cells in the G0/G1 (in blue), S (in red), G2/M (in dark green) phases of the cell cycle and represent the mean ± SEM of three independent experiments; ^a,b,c,d^ show significant differences between different groups (*p* < 0.05).

The effect of AME exposure on different phases of the cell cycle is shown in Figure 3. AME induces a dose-dependent increase in the percentage of cells in the G2 phase. Compared to the untreated control, AME has induced a significant increase in the percentage of cells in the G2 phase for concentrations of 2.5 μM (33.59%) and 5 μM (41.9%) compared to the untreated control (19.43%). This increase in the percentage of the cells in the G2 phase induced by the tested concentrations of AME (2.5 and 5 μM) was negatively correlated with 1.19- and 1.33-fold decreases in both the percentage of the cells in the G0/G1 phase and the percentage of the cells in the S phase by 1.7- and 1.98-fold compared to the control. Except causing a small decrease in the cell count percentage in the GO/G1 phase, a concentration of 0.5 μM does not have any other effect on the phases of the cell cycle.

### 2.3. Effect of AME on Caspase 3/7 Activation

**Figure 4:**
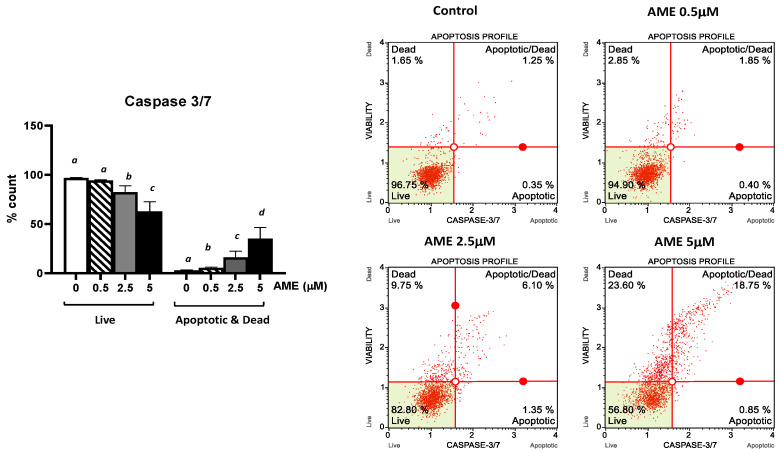
Effect of AME mycotoxin on caspase 3/7 activation. IPEC-1 cells were treated or left untreated for 24 h with different concentrations of AME, and the apoptotic status based on caspase-3/7 activation as well as cellular plasma membrane permeabilization and cell death was analyzed using the Muse Caspase-3/7 kit, according to the manufacturer’s instructions. Data are presented as a percentage of cells in various stages of apoptosis based on Caspase-3/7 activity and represent the mean ± SEM of three independent experiments; ^a,b,c,d^ show significant differences between different groups (*p* < 0.05).

Further, we investigated the apoptotic status based on caspase-3/7 activation as well as cellular plasma membrane permeabilization and cell death induced by the exposure of the cells to AME for 24 h (Figure 4).

As expected, AME induces a dose-dependent increase in the percentage of apoptotic cells. This increase was more important for higher concentrations of AME: 8.73% for 2.5 μM and 19.23% for 5 mM, respectively. These results show that the activation of executioner caspase 3/7 has an important role in the apoptotic process triggered by AME.

### 2.4. Effect of AME on Antioxidant Enzyme Activity

The activities of the antioxidant enzymes glutathione peroxidase, catalase, and superoxide dismutase were analyzed in IPEC-1 cells exposed to the AME mycotoxin.

**Figure 5:**
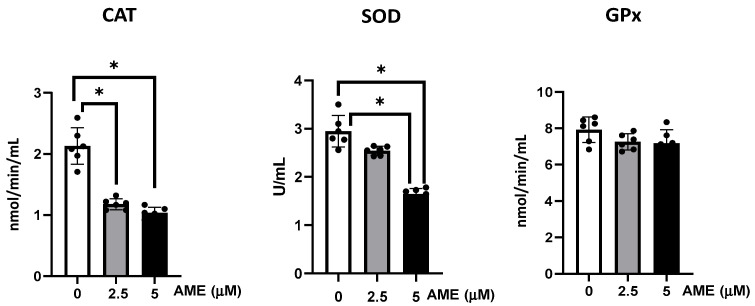
Effect of AME mycotoxin on the activity of the enzymes involved in oxidative stress. The activity of catalase (CAT), superoxide dismutase (SOD), and glutathione peroxidase (GPx) were assessed in IPEC-1 cells that were treated or left untreated for 24 h with AME. Data represent the mean ± SEM of three independent experiments; * represents significant differences between different groups (* *p* < 0.05).

As shown in Figure 5, AME interfered with the activity of the all the analyzed antioxidants, but significant effects were obtained only for catalase and superoxide dismutase. Thus, the activity of catalase was significantly decreased by 44.7% at a 2.5 μM AME concentration and by 51.3% at a 5 μM AME concentration compared to the untreated control. The activity of superoxide dismutase also decreased by 13.7% at a 2.5 μM AME concentration and by 44.2% at a 5 μM AME concentration.

### 2.5. Effect of AME on Markers of Oxidative Stress

**Figure 6:**
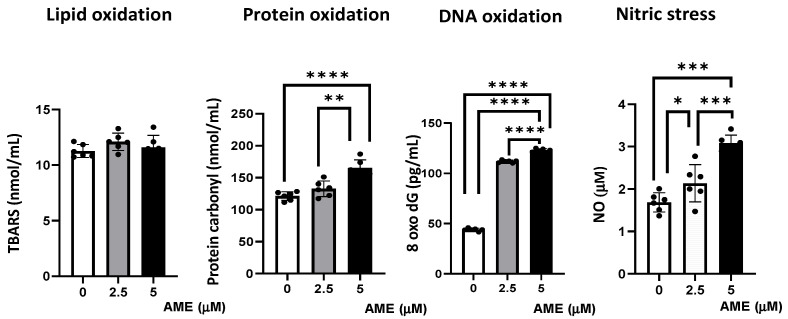
Effect of AME mycotoxin on different parameters associated with oxidative stress. Lipid oxidation (TBARS), protein oxidation (protein carbonyl), DNA oxidation (8 oxo dG), and nitrosative stress (NO) were assessed in IPEC-1 cells that were treated or left untreated for 24 h with AME. Data represent the mean ± SEM of three independent experiments; asterisks represent significant differences between different groups (* *p* < 0.05; ** *p* < 0.01; *** *p* < 0.001; **** *p* < 0.0001).

The capacity of AME to induce oxidative stress was analyzed by assessing lipid, protein, and DNA oxidation (Figure 6).

Compared to the untreated control, AME induced an increase in protein oxidation, which resulted from the significant increase in the concentration of protein carbonyl in cells exposed to a 2.5 μM AME concentration (9.4% increase; *p* = 0.0718) and a 5 μM AME concentration (36.16% increase; *p* < 0.0001). Also, the concentration of 8-Oxo-2′-deoxyguanosine, similarly to the major products of DNA oxidation, increased in cells exposed for 24 h to a 2.5 μM AME concentration by 2.55 times and by 2.80 times when subjected to a 5 μM AME concentration. The concentration of nitric oxide as a marker of nitrosative stress also increased significantly after the exposure of cells to the toxin (26.79% increase at a 2.5 μM concentration; 60.16% increase at a 5 μM concentration). Moreover, the level of thiobarbituric acid reactive substances/malodialdehyde (TBARS/MDA), which are markers of lipid peroxidation that are induced by oxidative stress, was not affected by the exposure of IPEC-1 cells to AME.

### 2.6. Effect of AME on Several Markers’ Gene Expression of Cell Signaling Pathways Involved in Oxidative Stress

**Figure 7:**
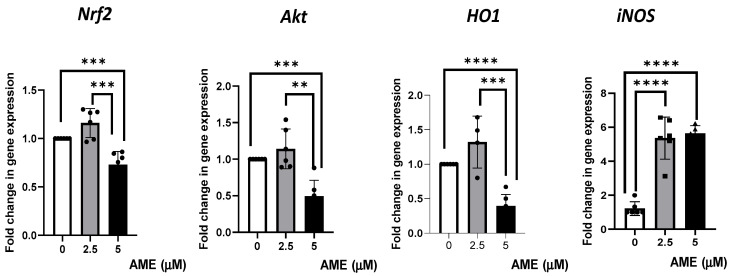
Effect of AME mycotoxin on several markers’ gene expressions of cell signaling pathways involved in oxidative stress. Gene expression of target genes Nrf2, Akt, Ho1, and iNOS was assessed in IPEC-1 cells that were treated or left untreated for 24 h with AME. Data were normalized to the geometric mean of two reference genes and expressed as fold changes (Fc), and they represent mean ± SEM of three independent experiments, with duplicate samples. Asterisks shows significant differences between different groups (** *p* < 0.01; *** *p* < 0.001; **** *p* < 0.0001).

Then, we investigated the effects produced by AME on several markers’ gene expression of cell signaling pathways involved in oxidative stress (Figure 7).

The results obtained from the qPCR analysis showed a slight, unsignificant increase in gene expression for Nrf2, Akt, and HO1 after treating the cells with 2.5 μM of AME. The highest concentration of AME (5 μM) induced a significant decrease in Nrf2, Akt, and HO1 by 0.27% (*p* = 0.0007), 0.51% (*p* = 0.0002), 0.61% (*p* < 0.0001), respectively, compared to the control. AME was responsible for a significant increase in iNOS, regardless of the AME concentration used (4.39-fold increase for a 2.5 μM concentration and a 4.62-fold increase for a 5 μM concentration compared to the control).

## 3. Discussion

Analyses of feed and feed ingredient samples collected in Europe have shown frequent contamination with *Alternaria* toxins, especially with AOH, AME, and TeA [12]. Consumption of contaminated feed could give rise to health problems in livestock. In particular, swine are very sensitive to mycotoxin toxicity [9]. The diet of swine is rich in cereals, and recent studies have shown that unprocessed cereals can be contaminated with *Alternaria* toxins [25]. For example, AME was found in cereals in a range between 0.3 and 905 µg/kg [26]. Although the contamination of feed and feed ingredients is well documented, studies carrying out risk assessments in swine are still lacking.

The gut represents the first barrier between the organism and the environment; thus, it is very important to know how gut epithelial cells respond when they are exposed to mycotoxins.

The aim of the present study was to analyze the effects of alternariol monomethyl ether—a frequent contaminant of swine feed—on pig intestinal health using an epithelial intestinal cell line model: IPEC-1. The use of intestinal epithelial cell models can provide, with some limitations, important information for assessing the effects of different compounds, including toxins, at the gut level [17]. Although an in vitro study cannot replace an in vivo study, it can provide a partial image of the toxicological effects that can occur at the intestinal level after the ingestion of a feed contaminated with AME. However, the use of cell models for toxicological studies is frequent, and our study aimed to identify the effects of AME on the intestinal epithelial cells of porcine origin (IPEC-1) as the information concerning the toxic effects of *Alternaria* toxins is scarce, including information on in vitro studies. The in vitro cell model used in this study was represented by proliferating cells, as (at the intestinal level) epithelial cells are continuously renewed and can interact with different xenobiotics, including mycotoxins [27]. Thus, this represents a realistic scenario in which cells are exposed to the AME mycotoxin. In our study, IPEC-1 cells were exposed to the toxin for 24 h, which is a realistic time period for it to reach the gut, be absorbed, and exert cytotoxic effects. We utilized this timer interval as it previously proven that 24 to 96 h is needed for the passage of solid feed through pigs’ entire gastro-intestinal tract [28].

Previous studies have shown that *Alternaria* toxins can induce a decrease in intestinal epithelial cell proliferation and cell viability [20,29]. However, most of the studies have evaluated the cytotoxic effect of AOH and/or using transformed cell lines, such as colorectal carcinoma human cells. The few studies that have analyzed the cytotoxic effect of AME on intestinal epithelial cell proliferation have shown that the inhibition of cell growth is dose- and time-dependent for a toxin within the range of 25–100 μM in CaCo2 cells, or within 10–200 μM in HCT116 cells [30,31]. The IC50 value calculated for the HCT116 cell line was equal to a 120 μM concentration [30,32]. Our results have also shown a dose-related effect on cell viability, but the IC50 value was lower (10.5 μM) than in the case of transformed cell lines; this could be related to the fact that IPEC-1 is a primary line and because the cells in our study were not differentiated, making them more sensitive to the toxic effects of a mycotoxin in comparison to modified and differentiated cells.

A decrease in cell proliferation following the cells’ exposure to *Alternaria* toxins was usually accompanied by the arrest of cells in the G2/M-phase [33,34,35,36], although an increase in the percentage of cells in the G1 or sub-G1 phases was also reported in some other cell types, such as human hepatocarcinoma cells, human colon carcinoma cells, and swine porcine endometrial cells [32,37,38]. Alternariol induces DNA strand breaks as it acts as a topoisomerase poison, especially for the topoisomerase II alpha isoform, with subsequent cycle arrest at the G2/M phase, p53 activation, and increased expression of p21, Cyclin B, MDM2, and sestrin 2 [36,39]. Previous studies have also shown that the exposure to AME leads to DNA damage and cell cycle arrest [30,34]. Our findings also indicate a significant increase in the percentage of cells in the G2 phase as well as a decrease in the percentage of cells in the G0/G1 and S phases induced at concentrations of 2.5 μM AME and 5 μM AME compared to the untreated control.

Severe DNA damage caused by the exposure to AME can lead to the transition from cell cycle arrest to apoptosis through the mitochondrial pathway [30]. In our study, compared to the control cells, the percentage of necrotic dead cells at the highest concentrations of the toxins used in the apoptosis study increased 4.8-fold compared to the control, while the percentage of total apoptotic cells (early + late apoptotic cells) increased 32.5-fold, showing that the toxin induces programmed cell death through apoptosis rather than a death via the necrosis of IPEC-1 cells. AME induces the activation of the mitochondria-dependent “intrinsic” apoptotic pathway in human colon carcinoma cells through mitochondrial membrane permeabilization and cytochrome c release, which initiates the activation of a proteolytic cascade of caspases, leading to apoptosis [30]. Our data has also indicated a dose-related increase in the apoptotic status based on caspase-3/7 activation induced by AME in IPEC-1 cells.

Different pro-apoptotic factors, such as p53, act on mitochondria, which is the central executioner of programmed cell death, to induce oxidative stress [40]. Reactive oxygen species (ROS) produced in excess induce the opening of the mitochondrial permeability transition pore, followed by a release of apoptogenic signaling molecules [41].

Bensassi and collaborators have shown that *Alternaria* toxins can induce an opening of the permeability transition pore, triggering apoptosis through the mitochondrial pathway [32]. A few studies have indicated an involvement of AME in triggering oxidative stress as the exposure to the toxin can increase reactive oxygen species (ROS) in human esophageal cells [42]. Generally, an overproduction of ROS induces irreversible damage to DNA, lipids, and proteins [43]. In IPEC-1 cells exposed to AME (2.5 μM and 5 μM), there was a significant increase in the concentration of protein carbonyl, which is a marker of protein oxidation, and of the concentration of 8-Oxo-2′-deoxyguanosine, which is the major product of DNA oxidation. Similarly, Solhaug and collaborators have shown that AOH exposure induced an increase in oxidative DNA damage, which was correlated with an increase in ROS levels and alterations in cellular pathways related to genomic integrity, apoptosis, and mitochondrial damage [36]. Additionally, our data have shown that AME interferes with the antioxidant response by significant decreasing the enzymatic activity of catalase and superoxide dismutase in IPEC-1 cells. Other *Alternaria* toxins, such as alternariol, were also associated with the generation of ROS, and they impaired antioxidant enzymatic defenses and the glutathione protective mechanism in in vitro studies [44].

The expression of antioxidant enzymes is mainly regulated by the nuclear erythroid 2-related factor 2 (Nrf2), a transcription factor that controls the redox environment by regulating the cellular defense mechanisms against oxidative and toxic stresses [45]. Activation or blockage of the Nrf2 pathway is considered one of the potential mechanisms involved in mycotoxin toxicity in humans and animals [46,47].

At a low concentration, the activation of Akt/Nrf2/HO-1 signaling would be beneficial for protecting IPEC-1 cells from the oxidative stress induced by AME. Indeed, AME was shown to modulate the cellular redox status via the activation of the redox-sensitive Nrf2/ARE pathway [48]. In this study, a short exposure time (3 h of incubation) to AME was not able to induce DNA damage, indicating that the DNA repair process was enhanced [48]. However, in our study, the exposure of IPEC-1 cells for 24 h to a 5 μM AME concentration induced a significant decrease in Nrf2, Akt, and HO1 gene expression but also a significant increase in DNA oxidation in IPEC-1 cells compared to the control. A decrease in Nrf2 expression accompanied by a decrease in phase II detoxifying enzymes HO-1, glutamate-cysteine ligase regulatory subunits (GCLM), and NAD(P)H quinone dehydrogenase 1 (NQO1) was also found in prostate epithelial cells as a consequence of exposure to alternariol at a concentration of 10 μM [49].

Nitric oxide is an important signaling molecule that can be endogenously generated in cells, and it is involved in several biological processes, such as neurotransmission, vasodilation, inflammation, apoptosis, and tumoral growth [50]. In our study, the concentration of nitric oxide increased significantly after the exposure of cells to the toxin, and it correlated with an increase in iNOS gene expression. In tobacco BY-2 cells exposed to *Alternaria* toxins, the combined effects of NO and ROS were required for cell death. On the other hand, in the presence of ROS, the autophagy process was initiated [51]. Solhaug and collaborators have also found that AOH induces autophagy in the RAW264.7 macrophage cell line through the Sestrin2-AMPK-mTOR-S6K pathway and that AOH-induced sestrin 2 expression can be inhibited by antioxidants [52].

**Figure 8:**
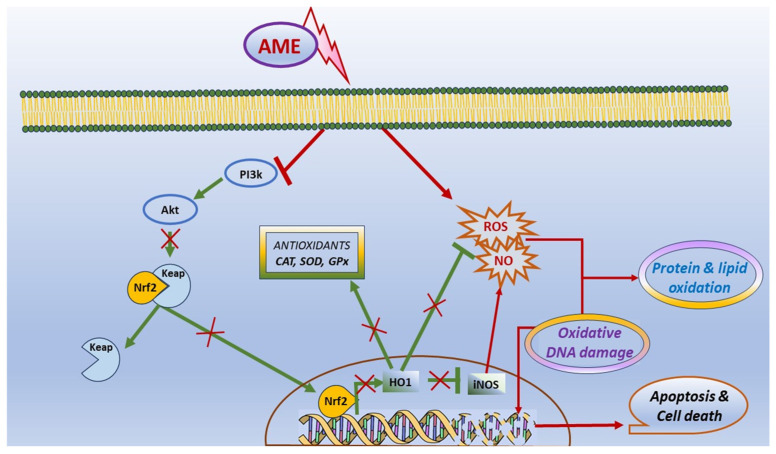
Scheme illustrating the effect of AME on oxidative stress and apoptosis.

In conclusion, our results have shown that the exposure to AME can interfere with cell viability, apoptosis, and death in IPEC-1 cells, and it was correlated with the triggering of oxidative stress. Briefly, we found that the exposure of porcine epithelial intestinal cells IPEC-1 to AME significantly decreased cell proliferation, inducing an alteration in cell cycle. Moreover, we observed a significant increase in the percentage of cells in the G2 phase and an increase in total apoptotic cells, suggesting that AME induces programmed cell death through apoptosis based on caspase-3/7 activation. AME induced oxidative stress in IPEC-1 cells through DNA and protein oxidation and decreased the activity of antioxidant enzymes catalase and superoxide dismutase. These effects on the oxidative response can be related to an inhibition of the Akt/Nrf2/HO-1 signaling pathway and are summarized in Figure 8. Further studies are needed in order to validate these in vitro data using in vivo trials in swine.

## 4. Material and Methods

### 4.1. Reagents

Unless otherwise specified, all the reagents were purchased from Sigma Aldrich (St. Louis, MO, USA).

### 4.2. Cell Culture and Toxin

Intestinal porcine epithelial cell lines (IPEC-1) between passages 122 and 133 were seeded in 24-well cell culture plates at a concentration of 2 × 10^5^ cells/mL and grown in a humidified CO_2_ incubator in complete DMEM/F-12 medium supplemented with a mixture of antibiotics (penicillin 100 UI/mL and streptomycin 50 µg/mL), 5% fetal bovine serum, 2 mM L-glutamine, 5 µg/L epidermal growth factor, ITS: 10 µg/mL insulin, 5 µg/mL transferrin, and 5 ng/mL sodium selenite (Gibco, Thermo Fisher Waltham, MA, USA). After 24 h, cells were treated or left untreated with AME for another 24 h and used for assessing the parameters related to cell proliferation, apoptosis, and oxidative stress.

### 4.3. MTT Assay

Cell cytotoxicity was measured with MTT [3-(4,5-dimethylthiazol-2-yl)-2,5-diphenyl tetrazolium bromide] assay, as previously described [53], for AME concentrations of 0.5, 2.5, 5, 25, 50, and 100 μM. Cell viability was assessed according to the manufacturer’s instructions after incubation. Cell viability was expressed as the percentage of the control cell. All tests were performed in three independent experiments.

### 4.4. Flow Cytometry Analyses

For flow cytometry analyses, cells were exposed to AME concentrations of 0.5, 2.5, 5, 25, and 50 μM based on the previous results obtained in MTT assay. Muse Cell Analyzer system and Muse 1.5 Analysis software (Merck, Darmstadt, Germany) were used for performing flow cytometry analyses using Muse kits for cell cycle, apoptosis, and cell signaling in apoptosis (Annexin and Dead Cell kit, Cell Cycle Analysis kit, Caspase 3/7 kit, Bcl-2 Activation Dual Detection kit) according to the manufacturer’s instructions, and as already described in our previous studies [54]. The results are expressed as percentages of cells and represent the mean of three independent experiments. Doses lower than IC50 were used in the experiment assessing the AME effect on oxidative stress.

### 4.5. Antioxidant Enzyme Activity

The activities of the antioxidant enzymes glutathione peroxidase (GPx), catalase (CAT), and superoxide dismutase (SOD) were analyzed using Cayman Chemical Company kits (Ann Arbor, MI, USA) from IPEC-1 cell lysates, as described by [9]. Briefly, the samples were mixed with the working solutions according to the manufacturer’s instructions, and the absorbances were read using a microplate reader (Tecan, Sunrise, Vienna, Austria). The results for GPx and CAT were expressed in nmol/min/mL, with SOD activity being reported in U/mL. The results represent the means of three independent experiments.

### 4.6. Assessment of Lipid Peroxidation

Lipid oxidation was determined from cell sample lysates in PBS, as already described [9]. Briefly, the protein concentrations of cell lysates were read using a NanoDrop and normalized to 1000 ng/mL protein for each sample. In total, 130 µL of cell lysates was mixed with 26 µL of HCL 0.5 N and 260 µL of TBA-TRIS and incubated for 60 min at 95 °C. The reaction was stopped by placing the samples on ice for 10 min, with the resulting absorbance being read using a microplate reader (Tecan, Sunrise, Vienna, Austria) at an absorbance of 532 nm. Results were expressed in nmol/mL malondialdehyde (MDA).

### 4.7. Assessment of Protein Oxidation

Protein oxidation was assessed using a spectrophotometric method based on the detection of the reaction product between 2,4-dinitrophenyl hydrazine and protein carbonyls. The protein concentrations from cell samples were determined using the Pierce BCA Protein Assay kit (ThermoFisher Scientific Rockford, IL, USA). The absorbances were determined at a wavelength of 370 nm using a microplate reader (Tecan, Sunrise, Vienna, Austria), with the results being expressed in nmol/mg of carbonyl content.

### 4.8. Assessment of DNA Oxidation

DNA oxidation was evaluated with the immune-enzymatic method using a DNA/RNA Oxidative Damage ELISA kit (Cayman Chemical Company, Ann Arbor, MI, USA). Briefly, 50 µL of DNA extracted from cell lysates was digested with nuclease P1 and mixed with 50 µL DNA/RNA Oxidative Damage AChE Tracer and 50 µL of DNA/RNA Oxidative Damage ELISA monoclonal antibody, which were provided in the kit. After 18h of incubation at 4 °C, the plate was washed and developed using 200 µL/well of Ellman’s Reagent, which was provided in the kit. The absorbances were read at a wavelength of 420 nm, and the results for each sample were expressed in pg/mL of 8-hydroxy-2′-deoxyguanosine.

### 4.9. Nitric Oxide Concentration

Griess assay was used for the quantification of the NO concentration in plasma, as already described in our previous study [55]. Briefly, 80 µL of each sample was mixed with 10 µL nitrate reductase co-factor and 10 µL Nitrate Reductase enzyme. After 3 h of incubation in the dark at room temperature, 100 µL/well of a 1:1 mixture of Griess Reagent [(1% Sulphanilamide, 5% Phosphoric Acid): NED Reagent (0.1%)] was dispensed. The plate was incubated for 10 min at room temperature, and then the absorbance was read at 540 nm using a microplate reader (Tecan, Sunrise, Vienna, Austria).

### 4.10. Quantification of Gene Expression

The effects produced by AME on several markers’ gene expression of cell signaling pathways involved in the oxidative stress were investigated in IPEC cells exposed for 24 h to AME. Total RNA was extracted using a Qiagen RNeasy mini kit (Qiagen GmbH, Hilden, Germany). The concentration and the quality of the extracted RNA were evaluated using RNA ScreenTape Analysis kit (Agilent Technologies, Santa Clara, CA, USA) and a bioanalyzer (2100 Agilent Bioanalyzer, Agilent Technologies, Santa Clara, CA, USA). Further, cDNA was obtained from the revers transcription of mRNA using a M-MuLV reverse transcriptase kit (Thermo Fischer Scientific, Waltham, MA, USA). Quantitative PCR was used for the evaluation of the gene expression of several molecules involved in the response to oxidative stress, such as Nrf-2 (nuclear factor erythroid 2-related factor 2), Akt (a serine/threonine protein kinase), HO-1 (hem oxygenase), and iNOS (inducible nitric oxide synthase), using primer sequences and a qPCR protocol, as already described in our previous studies [56]. The changes in gene expression were assessed after the normalization of qPCR data using two reference genes—hypoxanthine-guanine phosphoribosyl transferase (HGPRT) and glyceraldehyde-3-phosphate dehydrogenase (GAPDH)—which selected from a total of six reference genes using NormFinder software (https://www.moma.dk/software/normfinder accessed on 18 December 2023). Gene expression was calculated using the 2^(−DDCT)^ method, and the results were presented as relative fold change (Fc) compared to control.

### 4.11. Statistical Analysis

Data of the results are expressed as means and standard errors. The IC50 value was calculated using GraphPad Prism 9 software. Differences among experimental groups were analyzed for significance using GraphPad Prism 9 software with one-way ANOVA followed by a Fisher PSLD test, and the differences between the experimental groups were considered significant at a *p*-value of < 0.05.

## Data Availability

Data can be made available upon request.
